# InDel markers: An extended marker resource for molecular breeding in chickpea

**DOI:** 10.1371/journal.pone.0213999

**Published:** 2019-03-18

**Authors:** Ankit Jain, Manish Roorkiwal, Sandip Kale, Vanika Garg, Ramakrishna Yadala, Rajeev K. Varshney

**Affiliations:** 1 International Crops Research Institute for the Semi-Arid Tropics (ICRISAT), Hyderabad, India; 2 Leibniz-Institut für Pflanzengenetik und Kulturpflanzenforschung (IPK), Gatersleben, Germany; National Institute for Plant Genome Research, INDIA

## Abstract

Chickpea is one of the most important food legumes that holds the key to meet rising global food and nutritional demand. In order to deploy molecular breeding approaches in crop improvement programs, user friendly and cost effective marker resources remain prerequisite. The advent of next generation sequencing (NGS) technology has resulted in the generation of several thousands of markers as part of several large scale genome sequencing and re-sequencing initiatives. Very recently, PCR based Insertion-deletions (InDels) are becoming a popular gel based genotyping solution because of their co-dominant, inexpensive, and highly polymorphic nature. With an objective to expand marker resources for genomics assisted breeding (GAB) in chickpea, whole genome re-sequencing data generated on five parental lines of one interspecific (ICC 4958 × PI 489777) and two intra-specific (ICC 283 × ICC 8261 and ICC 4958 × ICC 1882) mapping populations, were used for identification of InDels. A total of 231,658 InDels were identified using Dindel software with default parameters. Further, a total of 8,307 InDels with ≥20 bp size were selected for development of gel based markers, of which primers could be designed for 7,523 (90.56%) markers. On average, markers appeared at a frequency of 1,038 InDels/LG with a maximum number of markers on CaLG04 (1,952 InDels) and minimum on CaLG08 (360 InDels). In order to validate these InDels, a total of 423 primer pairs were randomly selected and tested on the selected parental lines. A high amplification rate of 80% was observed ranging from 46.06 to 58.01% polymorphism rate across parents on 3% agarose gel. This study clearly reflects the usefulness of available sequence data for the development of genome-wide InDels in chickpea that can further contribute and accelerate a wide range of genetic and molecular breeding activities in chickpea.

## Introduction

Chickpea (*Cicer arietinum* L.) is a self-pollinated crop with a basic chromosome number eight and ~740 Mbp genome size [1]. Chickpea is predominently grown on low input marginal lands of arid and semi-arid regions [2]. It is considered as an important component of subsistence farming in developing countries especially to resource poor farmers. A well balanced nutritional food with 20–30% protein, ~40% carbohydrates, minerals, vitamins, soluble and insoluble fiber, chickpea is an ideal human diet and animal feed, thus plays a significant role in food and nutritional security globally [3]. Like other legumes, chickpea is also known to symbiotically fix atmospheric nitrogen with rhizobia, thus improving the soil health which makes it ideal for crop rotation programs [4]. It is estimated that, chickpea can fix up to 140 Kg N ha^-1^, thus minimizing the application of additional Nitrogen fertilizer in the field [5].

Currently, chickpea is being grown across 55 nations with an acreage area over 14.56 million hectares resulting in an annual yield of 14.78 million tonnes (FAO 2017). About 1 t ha^-1^ average productivity falls far below the actual potential (6 t ha^-1^) of the crop when grown under optimal conditions. Variable abiotic stresses such as temperature, drought, salinity, and biotic factors such as Fusarium wilt (FW) caused by *Fusarium oxysporum* f.sp. *ciceri* and Ascochyta blight (AB) caused by *Ascochyta rabiei* (Pass.), are major factors contributing to productivity losses in chickpea [6]. Among abiotic stresses, terminal drought is a major production constraint as it delays flowering and affects seed yield. Drought alone is estimated to reduce yield in chickpea by 33% annually [7] and is expected to become more severe under predicted climate change scenarios. Therefore, there is a dire need to develop improved chickpea varieties that can withstand various biotic and abiotic stresses.

Deployment of genomics assisted breeding (GAB), that is the integration of genomic approaches in breeding, is a powerful approach in enhancing crop productivity [8,9]. Thus, it is imperative to identify and further utilize genomic regions/genes/alleles that are responsible for higher crop productivity using GAB technologies. Until last decade, application of GAB approaches had been a challenging task because of the meagre availability of genomic resources, making chickpea an orphan crop. However, the last decade has witnessed a tremendous increase in the availability of genomic resources to harness the variability of germplasm resources. A shift from isozyme and random amplified polymorphic DNA (RAPD) to amplified fragment length polymorphism (AFLP), simple sequence repeat (SSRs), and single nucleotide polymorphisms (SNPs) has occurred and these markers have been applied in a variety of scenarios ranging from diversity studies to genetic maps construction and QTL analysis for some of the most important agronomic traits [10]. The advent of next-generation sequencing (NGS) and high-throughput genotyping technologies has reduced the genotyping and sequencing cost drastically, which enabled the availability of a draft genome, further enhancing the depth of understanding and extending the genomic resources in chickpea [1]. NGS based technologies have resulted in the availability of large genomic resources and enriched the marker repository. However, there is still a need to validate these *in silico* resources and develop markers that can be efficiently used with limited infrastructure requirements. In the recent past, polymorphism attributed by PCR based InDels have received more attention because of their co-dominant inheritance, reproducibility and easy to use nature [11].

InDels are structural variations distributed abundantly throughout the genome, arising as a result of polymerase slippage, transposons, unequal crossing-over etc., that may sometimes lead to the gain/loss of function in the organism [12–15]. The most common categories of InDels involve single base pair insertion and deletion, monomeric base pair expansion and multi base pair expansion [11,16–17]. However, InDels containing random sequences and transposon insertions are comparatively less prevalent among genomes [11]. InDels are being used in a variety of applications including population genetics, taxon diagnostic markers, genetic map construction and association mapping in different crop plants *viz*. rice (*Oryza sativa* L.) [18], Arabidopsis (*Arabidopsis thaliana*) [19], barley (*Hordeum vulgare*) [20], tomato (*Solanum lycopersicum* L.) [21], pepper (*Capsicum annuum*) [22], Phaseolus (*Phaseolus vulgaris* L.) [23] and Brassica (*Brassica rapa*) [24] etc. Further, as InDels can be genotyped with simple gel based size separation procedures and the absence of stutter bands makes InDels more valuable. In some of the previous studies, InDels were also found to be more polymorphic than microsatellite markers [24,25]. The availability of the whole genome sequence of chickpea [1, 26, 27] has resulted in vast genome information and further paved the way for various large scale resequencing initiatives ([28,29], unpublished data) making it easy to capture the variation existing among genotypes.

With an objective to enhance the marker repertoire and develop the breeder friendly markers with limited infrastructure requirements in chickpea, the current study focuses on identification and development of InDels in five chickpea parental lines. Some of these randomly selected markers have been validated for their efficacy on agarose gel electrophoresis.

## Materials and methods

### DNA isolation

A high throughput mini-DNA extraction method was standardized, with certain modifications from an earlier method [30]. In brief, the method involved following steps: harvested leaves from 15 days old seedlings, were grounded using steel balls in preheated (65°C) CTAB buffer (100 mM Tris-HCl (pH-8), 1.4 M NaCl, 20 mM EDTA, CTAB (2–3% w/v)) with GenoGrinder (Spex CertiPrep, USA) at 1500 rpm for 2 mins. Ground samples with CTAB buffer were incubated for 10 mins at 65°C. After bringing CTAB buffer mixed grounded sample to room temperature, it was subjected to solvent extraction by mixing an equal volume of chloroform-isoamyl alcohol (24:1), followed by centrifugation at 5500 rpm for 10 mins. The aqueous phase was collected and DNA precipitation was done by adding 0.7 volume of isopropyl alcohol, and subject to a brief incubation at -80°C. DNA was precipitated by centrifugation of the the mixture at 5500 rpm for 10 mins. In order to purify the DNA, precipitated DNA was suspended in low TE buffer (10 mM Tris EDTA (pH-8)) and simultaneously was subjected to RNAse treatment (10 mg/ml) for 30 mins at 37°C. An equal volume of phenol-chloroform-isoamyl alcohol (25:24:1) was added to RNAse treated DNA and it was centrifuged. The aqueous phase collected was subject to DNA precipitation with 10% of Sodium acetate (3M NaOAc (pH-5.2)) and double volume of ethanol, followed by overnight incubation at -80°C. DNA was precipitated and washed with 70% ethanol. After drying at room temperature pellets were re-suspended in low-salt TE and stored at 4°C until further use. Estimation of quality and quantity of DNA was done using both agarose gel electrophoresis and spectrophotometer (Shimadzu UV160A, Japan).

### Resequencing and InDels screening

The whole genome re-sequencing (WGRS) data on selected five parental lines of chickpea inter- and intra-specific populations (ICC 1882, ICC 4958, ICC 283, ICC8261, PI 489777) generated as part of an earlier study [28] were used for InDels identification in the current study. The WGRS data for these parental lines were first aligned against the chickpea reference genome [1] using the BWA software and using default parameters [31]. The aligned data in BAM format were then used for searching InDels using Dindel software [32] with default parameters for diploid species. Briefly, DinDel first extracts all the indels from BAM file and group them in the window of 150 bp. It then identifys the candidate haplotypes and realing the reads around these candidate indels. Finally, it produces a vcf file with indel calls and qualities. The snpEff software was used for InDel annotation. In order to develop the cost effective gel based markers, InDels with size ≥20 bp were considered. Primers for the flanking region of the identified InDels were designed using Primer3 [33].

### PCR amplification

For validating identified InDels, PCR reaction mix was prepared for 10 μl volume consisting of DNA 10 ng, 0.1 U of *Taq* polymerase (Kappa), 5X Taq buffer with 25 mm MgCl_2_, 5 μM of forward and reverse primers, 2.5mM dNTPs. PCR amplifications were conducted with ABI thermal cycler (PE Applied Biosystems, CA) using a common series of touchdown PCR amplification thermal profile. A touch down PCR amplification thermal profile consisted of 3 min of initial denaturation cycle at 94°C, followed by 10 cycles of denaturation at 94°C for 20 sec, annealing at 60°C for 20 sec and extension at 72°C for 30 sec, with a 0.5 °C decrease per cycle followed by 40 cycles of denaturation, annealing at 55°C and extension for the same duration as before with a final extension at 72°C for 20 min. Amplicons were resolved on 3% agarose gel electrophoresis, visualized and documented under UV light using a gel documentation system (Alpha Innotech gel documentation system). On the basis of banding pattern of intraspecific (ICC 283 × ICC 8261 and ICC 4958 × ICC 1882) and interspecific parental lines (ICC 4958 × PI 489777) data were recorded.

## Results

### Sequence analysis and InDels identification

**Fig 1:**
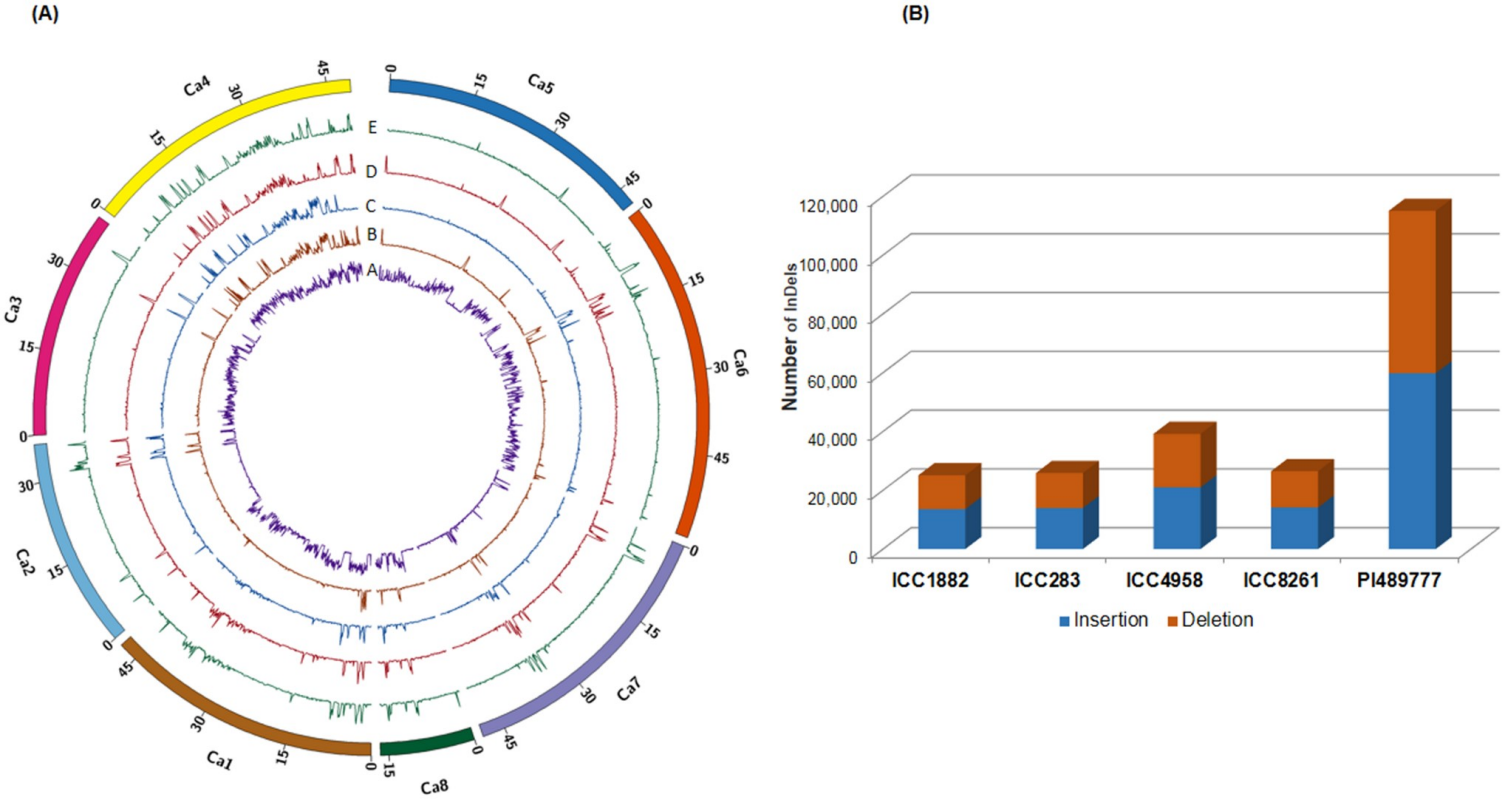
Distribution of InDels identified in five parental (A- ICC 1882, B- ICC 4958, C- ICC 283, D- ICC 8261 and E- PI 489777) along the eight linkage groups of chickpea. (A) Circular representation of the distribution of insertions and deletions in the chickpea genome. (B) Comparative distribution of insertion and deletions among five parental lines.

**Fig 2:**
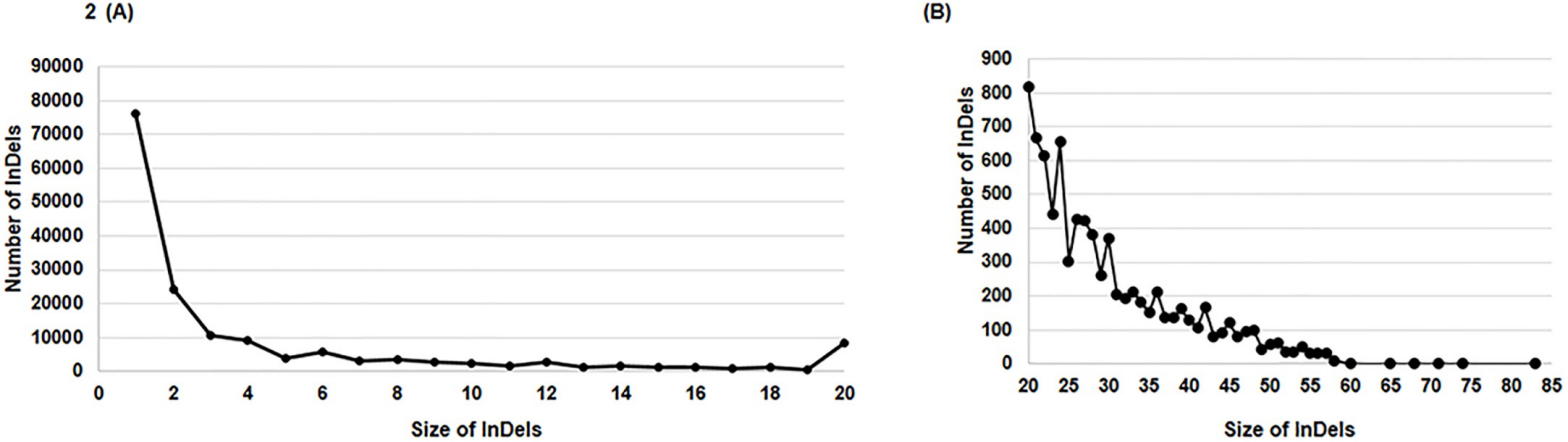
Relationship between InDels frequency and InDel lengths. (A) All length InDels frequency distribution in chickpea. (B) >20 bp InDels length distribution.

Resequencing data of five parental lines consisted of >106 million high quality reads with a minimum of 14.97 million reads for ICC 1882 to maximum of 43.57 million reads for ICC 4958. This accounted for 79.76% alignment of high quality reads with the reference genome. Sequence data showed an average genome coverage of 81.68%, with maximum for ICC 4958 (84.04%) and minimum for PI 489777 (79.76%) with mean depth ranging from 6.21 (ICC 1882) to 14.26 (ICC 4958) [28]. Screening of data with Dindel resulted in identification of a total 2,31,658 InDels across selected parental lines (Fig 1a). Of these identified InDels, 52.88% (1,22,512) were attributed to insertions and 47.12% (1,09,146) were attributed to deletions category (Table 1, Fig 1b). In total 1,61,784 (69.84%) unique InDels across these five different parents were identified. It was interesting to note that the range of insertions (51.97–54.18%) and deletions (45.82–48.02%) were not found to vary significantly among all the selected parental line individually. Among individual parental lines, maximum number of InDels (49.66%) were observed in PI 489777, the wild chickpea (*C*. *reticulatum*) accession, followed by ICC 4958 with 38,180 (16.91%), whereas abundance of InDels in remaining three parental lines was found almost in similar range i.e. 10.83% to 11.43% (Fig 1b, Table 1). With increase in InDels size, a decrease in abundance of InDels was observed (Fig 2a and 2b).

**Table 1:** Distribution of InDels across eight linkage groups of five parental lines.

Linkage group	ICC 1882			ICC 283			ICC 4958			ICC 8261			PI 489777		
	Insertions	Deletions	R_a_	Insertions	Deletions	R_a_	Insertions	Deletions	R_a_	Insertions	Deletions	R_a_	Insertions	Deletions	R_a_
**CaLG01**	2,351	2,016	0.090	2,103	1832	0.081	2,721	2,314	0.104	1,328	1,118	0.051	9,896	9,244	0.396
**CaLG02**	1,166	961	0.058	1,173	954	0.058	1,974	1,641	0.099	1,070	837	0.052	2,710	2,521	0.143
**CaLG03**	986	807	0.045	963	747	0.043	1,853	1,571	0.086	1,287	1,116	0.060	8,118	7,459	0.390
**CaLG04**	3,843	3,368	0.147	3,810	3405	0.147	6,771	6,175	0.263	5,421	4,977	0.211	14,553	13,332	0.567
**CaLG05**	926	761	0.035	1,206	1007	0.046	1,507	1,211	0.056	1,115	1,002	0.044	8,769	8,131	0.351
**CaLG06**	1,803	1,503	0.056	2,019	1743	0.063	2,966	2,487	0.092	1,923	1,660	0.060	11,294	10,543	0.367
**CaLG07**	1,860	1,539	0.069	2,057	1683	0.076	2,405	2,015	0.090	1,361	1,102	0.050	2,037	1,771	0.078
**CaLG08**	663	546	0.073	636	522	0.070	820	749	0.095	634	538	0.071	2,414	2,238	0.282
**Total**	13,598	11,501		13,967	11,893		21,017	18,163		14,139	12,350		59,791	55,239	

R_a_: Relative abundance of InDels

A large proportion of identified InDels was contributed by homozygous InDels (~91–97%) among parental lines except ICC 4958 where homozygous InDels accounted for competitively lower proportion (~54.5%) than rest of the parental lines (Table 2).

**Table 2:** Occurrence of homozygous and heterozygous InDels across five parental lines.

Genotype	Insertions		Deletions		Total
	Homozygous	Heterozygous	Homozygous	Heterozygous	
**ICC 1882**	12,833	765	10,864	637	25,099
**ICC 283**	13,181	786	11,249	644	25,860
**ICC 4958**	11,579	9,438	9,770	8,393	39,180
**ICC 8261**	13,013	1,126	11,113	1,237	26,489
**PI 489777**	57,916	1,875	53,238	2,001	1,15,030

### Establishment of PCR based InDels resource

**Fig 3:**
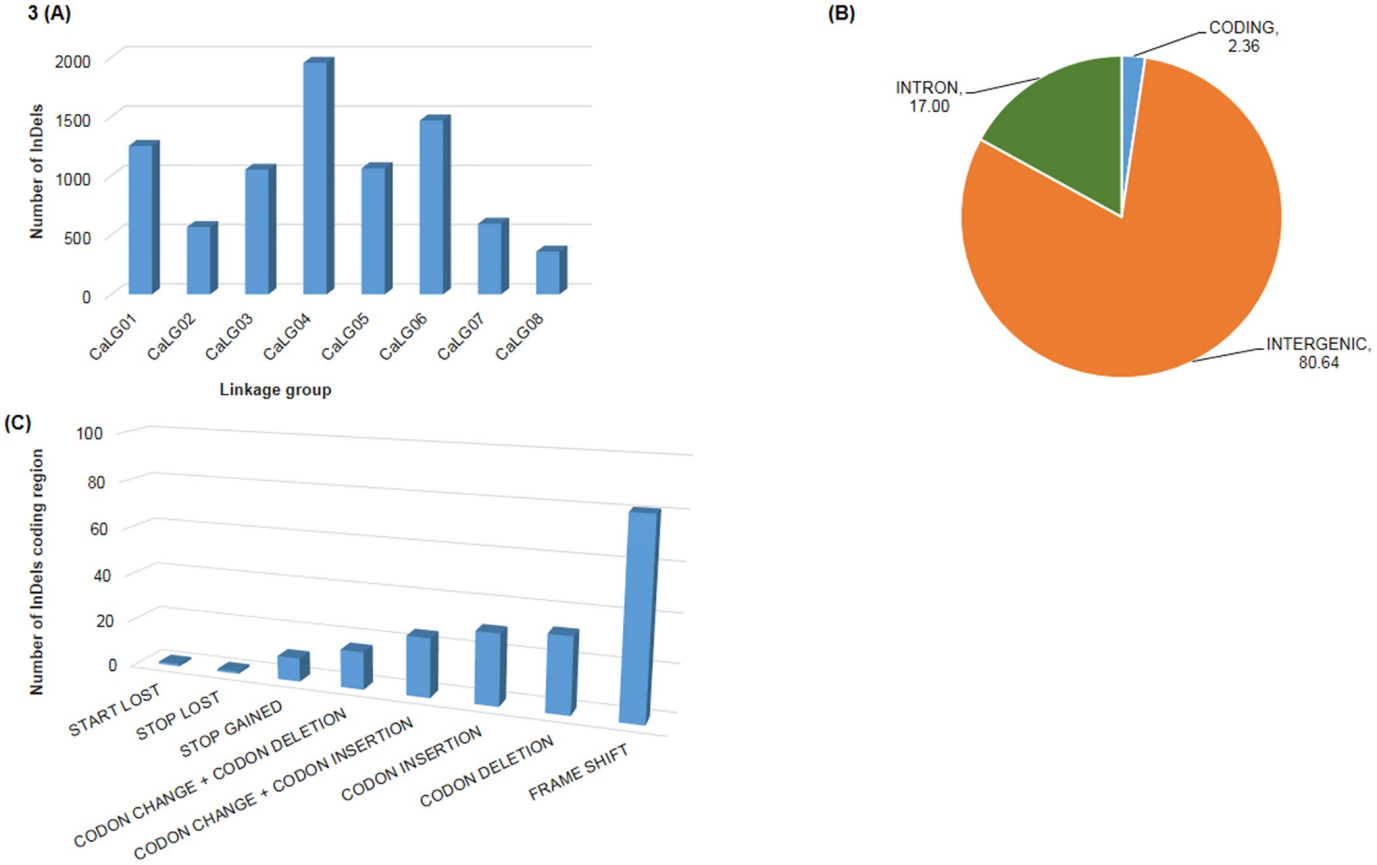
(A) Distribution of InDels (≥ 20 bp) across the eight linkage groups of chickpea. (B) Distribution of InDels in different genomic regions of Chickpea. (C) distribution of InDels in coding region of chickpea.

**Fig 4:**
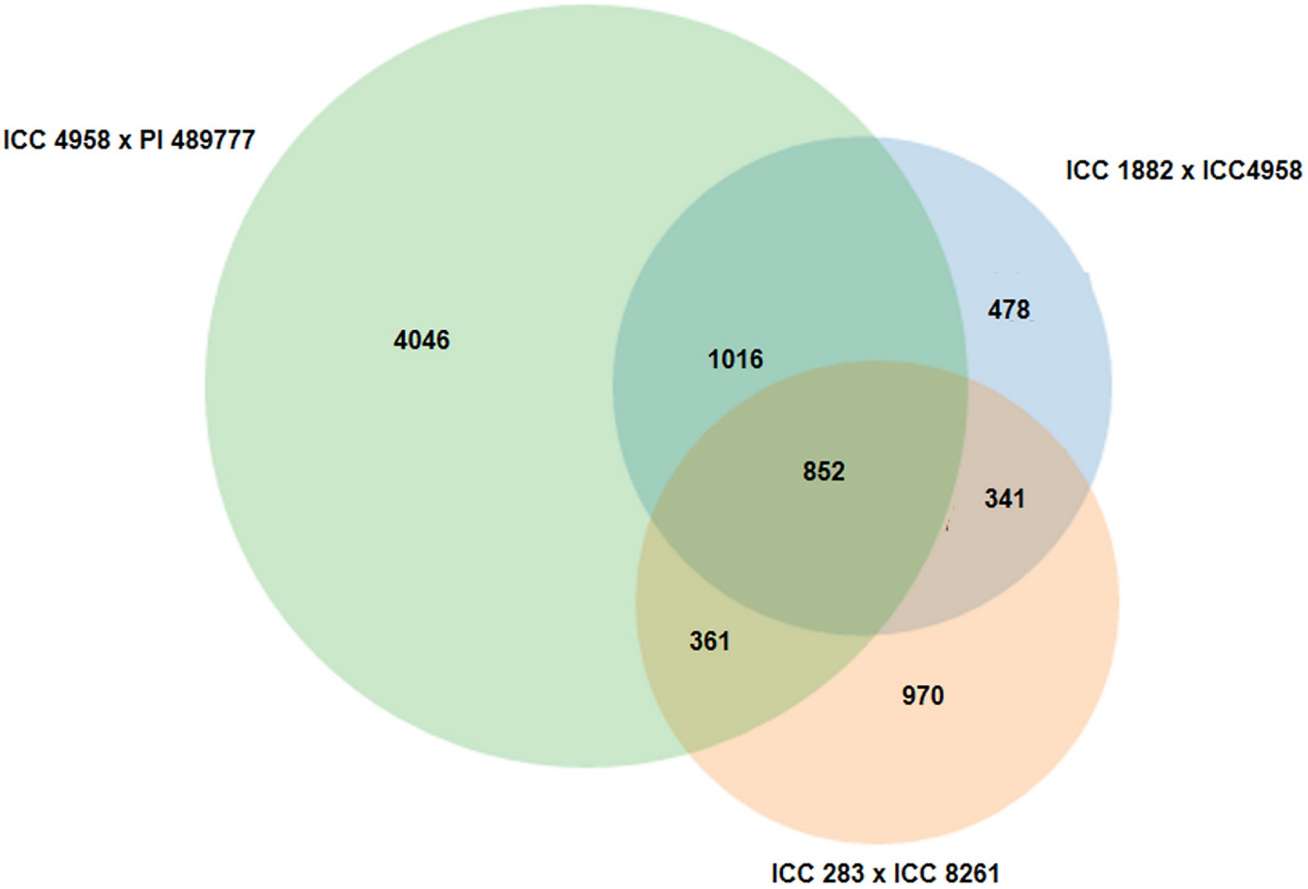
Venn diagram reflecting number of polymorphic InDels between interspecific (ICC 4958 × PI 489777) and intra-specific (ICC 283× ICC 8261 and ICC 4958 × ICC 1882) chickpea parental lines.

In order to establish a PCR based marker resource, we filtered out InDels with <20 bp size. As a result, 8,307 InDels with ≥ 20 bp size could be obtained. A higher abundance of InDels, was observed on CaLG04 with 1,952 InDels and minimum number was observed on CaLG08 with 360 InDels (Fig 3a). In total, 80.64% of InDels were observed in intergenic regions followed by 17% in intronic regions. Overall 2.36% of InDels were found to fall in coding region (Fig 3b and 3c). Based on length variations among InDels existing in parental lines, a total of 2,687 (32.35%) and 2,524 (30.38%) InDels were found to be polymorphic between ICC 4958 × ICC 1882 and ICC 283 × ICC 8261 respectively. Similarly, 6,275 (75.54%) InDels were identified as polymorphic between ICC 4958 and PI 489777. In total, 852 (10.26%) InDels were found to be polymorphic across all three populations undertaken in the study (Fig 4).

Primer pairs could be designed successfully for 7,523 (90.56%) InDels and are now available for the chickpea community as supplementary material for use in their respective chickpea programs (S1 Table).

### Validation of selected InDels

In order to validate the identified InDels, a total of 423 InDels were selected randomly and primer pairs were synthesized. The selected 423 primer pairs were used for amplification on five selected parental lines (S2 Table). Approximately 80% primer pairs resulted in successful amplification and amplicons were found producing expected band size on agarose gel. In total, 276 InDels were found to be polymorphic among the parental lines of inter-specific and intra-specific crosses. Overall, we observed 331 (ICC 4958 × PI 489777) to 343 (ICC 1882 × ICC 4958) primer pairs that yielded amplicons (excluding non-specific, multiple bands etc.) (Table 3). Based on amplification, a high polymorphic rate of 46.06% (158 InDels) and 56.43% (193 InDels) was observed between intra-specific parental lines and a polymorphic rate of 58.01% (192 InDels) was observed between parental lines of the inter-specific mapping population (Table 3). The maximum number of markers showing polymorphism between parental lines of the intra-specific populations namely ICC 283 × ICC 8261 and ICC 1882 × ICC 4958, came from CaLG01 with a polymorphic rate of 68% and 71.15% respectively. In the case of parental lines of ICC 4958 × PI 489777, maximum polymorphism rate was observed for InDels present on CaLG08 (85.71%) followed by InDels on CaLG01 that showed 78% polymorphism rate (Table 3). Validation results indicated InDels from CaLG06 attributed the least polymorphism rate (33.33%-43.08%) among intra-specific parental lines, whereas, for inter-specific parental lines, InDels from CaLG02 showed the minimum polymorphism rate (15.79%) (Table 3).

**Table 3:** Genome-wide distribution of InDels selected for validation and their performance in different mapping intra-specific and one inter-specific populations.

Linkage Group	Total markers	ICC 283 × ICC 8261			ICC 1882 × ICC 4958			ICC 4958 × PI 489777		
		Total amplified markers	Polymorphic markers	%	Total amplified markers	Polymorphic markers	%	Total amplified markers	Polymorphic markers	%
**CaLG01**	**58**	50	34	68.00	52	37	71.15	50	39	78.00
**CaLG02**	**42**	36	23	63.89	36	21	58.33	38	6	15.79
**CaLG03**	**61**	46	24	52.17	48	17	35.42	45	24	53.33
**CaLG04**	**65**	56	34	60.71	56	27	48.21	51	36	70.59
**CaLG05**	**52**	42	19	45.24	41	15	36.59	41	25	60.98
**CaLG06**	**81**	65	28	43.08	66	22	33.33	65	40	61.54
**CaLG07**	**45**	36	24	66.67	37	16	43.24	34	16	47.06
**CaLG08**	**19**	11	7	63.64	7	3	42.86	7	6	85.71
**Total**	**423**	**342**	**193**	**56.43**	**343**	**158**	**46.06**	**331**	**192**	**58.01**

## Discussion

In the era of NGS, a large number of genomic resources are being established. These resources have proven to be useful in enhancing genetic gains by implementation of GAB tools, and have successfully resulted in increased productivity in many crop plants [8,9]. In the case of chickpea, incorporation of molecular markers in improvement programmes has resulted in the development of some superior lines including lines with enhanced yield under rainfed conditions in JG 11 background [34], lines resistant to FW and AB in the genetic background of C 214 [35] and, lines resistant to FW in the genetic background of Pusa 256 [36]. Advanced molecular breeding approaches like genomic selection have already been initiated for yield related traits in chickpea [37,38]. In order to enhance the base of molecular markers available in chickpea, NGS technologies are being exploited and are aiding in the development of different types of user friendly markers across a vast range of applications [39,40]. Recently a study on flowering time in chickpea also identified one 11-bp deletion in the early flowering 3 (elf3) gene was found to be associated with early flowering in germplasm and successfully converted into KASP based InDel marker [41]. Although high-throughput genotyping platforms such as “Axiom *CicerSNP* Array” have become available [42], high infrastructure/costs and the specialized manpower requirements associated with these technologies keep them out of reach for low technology laboratories in many developing countries. However, low infrastructure laboratories can deploy PCR based markers for genotyping their germplasm.

Considering the high potential of InDels in comparison to SSRs and SNPs especially from the perspective of genotyping, we undertook resequencing data of five parental lines of inter-specific and intra-specific RIL populations and screened InDels. The maximum number of InDels were found on CaLG04 and minimum number of InDels were observed at CaLG08 in four parental lines except wild chickpea PI 489777, which showed the lowest frequency of InDels on CaLG07 (Table 1). This observation is supported by previous studies reporting a large number of variants (SNPs and/or InDel) on pseudomolecule CaLG04 [28,29,43,44]. As per our study InDel markers have shown higher rate of polymorphism as compared to SSR markers and almost similar level when compared with SNP markers. For instance, genetic mapping study based on using SSR on mapping population of these parental lines (ICC 4958 × ICC 1882 and ICC 8261 × ICC 283) showed polymorphism rate of <10% [43] however InDel markers based on parental polymorpshism in present study showed polymorphism rate of ~50% (Table 3). Similarly, genotyping on these SNP markers on these population (ICC 4958 × ICC 1882 and ICC 8261 × ICC 283) resulted in polymorphism rate of ~40% [42]. With an increase in InDels size, a decrease in abundance of InDels was observed (Fig 2a and 2b). A negative relationship was observed between the InDels size and abundance which has also been observed in previous studies in different crop plants [45]. In addition, the cost for genotyping using InDel markers is comparatively less and provide a cost effective approach for genotyping. For instance, several SNP genotyping service provider also provide genotyping at comparative cost with InDel markers, however SNP genotyping services are cost effective only in case are being used with large volumes of samples. However genotyping cost for InDel markers increase linearly with number of samples and can be undertaken with using commonly available equipment in lab [46]. It was interesting to observe that a decline in number of InDel loci with an increase in InDel length was not perfectly symmetrical.

Due to difference in the size of different linkage groups and to make parallel comparison of InDels across the eight linkage groups, the total number of InDels was normalized in order to assess relative abundance (R_a_) which is a measure of InDels distribution per Kb length of each linkage group (Table 1). Among all parental lines, CaLG04 continued to show maximum R_a_ than the rest of the linkage groups among different parental lines ranging from 0.147 InDels/Kb for ICC 1882 and ICC 283 to 0.567 InDels/Kb for PI 489777. Our results indicated that despite showing the minimum number of InDels on CaLG08, this linkage group did not account for the least R_a_ for any of the parental lines, which could be due to the smaller size of CaLG08 in comparison to other linkage groups. A high abundance of homozygous InDels was observed in the current study. Similar high abundance of homozygous InDels have also been observed for human genomes [47]. The huge abundance of homozygous InDels could be due to artifact, misclassification of heterozygous InDels possibly because of allele dropouts and heterozygous InDels being missed, which has also been observed in the case of humans by [47].

In order to resolve amplicons appropriately on agarose gels, InDels with ≥ 20 bp size were selected leading to a drastic reduction in the number of InDels. As an objective of this study, PCR based InDels were developed by identifying InDel sites and designing the primers for the flanking region. This led to the development of a resource with 7,523 primer pairs (S1 Table). Primers could not be designed for few InDels where primer designing criteria were not met.

InDels size differences existing among parental lines showed polymorphic potential of these markers in intra-specific and inter-specific RIL parental lines, which was further reaffirmed by validating randomly selected 423 primer pairs using agarose gel electrophoresis. Interestingly, some markers which were monomorphic in a particular population during *in silico* identification, were found to be polymorphic during PCR amplification and validation process. Such differences in polymorphism could be due to sequencing errors in the WGRS data which might have resulted in such InDel errors [21]. Similarly, reverse cases where InDels showing length difference *in silico* were found to be monomorphic during PCR and validation process were also observed. Such observations could be attributed to the incapability of gel based systems to resolve shorter InDels thus giving the impression as monomorphic markers. Non-amplification of certain primer pairs could be attributed to mismatch at primer site or existence of secondary structures of primers at annealing temperature leading to failed amplifications. Highest polymorphism was observed between the parental lines of the inter-specific population using both the PCR based approach as well as when comparing the InDels across the parental combinations, this can be explained by the more diverse genetic background of the wild and the cultivated line.

## Conclusion

The wealth of sequencing data generated using NGS technologies has resulted in the identification of millions of genome-wide markers. As the second most common variations after SNPs, InDels have the capability to affect/modify the function of genes. Unlike SNPs, InDels can be used in regular laboratories without much infrastructure and therefore can serve as a user friendly and cost effective marker system with a better polymorphic rate comparative to SSRs. The present study reports a repository of more than 7,000 potential InDel markers that might play an important role in different genetic studies and can be exploited for chickpea improvement through GAB approaches. Utility of these markers has also been established by using randomly selected 423 markers on 5 different chickpea accessions.

## Supporting information

S1 Table Detailed primer pair profile for InDels with ≥20 bp size. (XLSX)

S2 Table Polymorphism data for InDels validated using PCR amplification and gel electrophoresis, in five parental line combinations. (XLSX)
